# A comment on Morey et al. (2020)

**DOI:** 10.1515/tnsci-2020-0121

**Published:** 2020-06-15

**Authors:** Seth M. Levine

**Affiliations:** Department of Cognitive and Clinical Neuroscience, Central Institute of Mental Health, Medical Faculty Mannheim, Heidelberg University, 68159 Mannheim, Germany; Department of Psychiatry and Psychotherapy, University of Regensburg, 93053 Regensburg, Germany

Recent functional MRI studies have demonstrated that aversive learning can alter high-dimensional representations of conceptual information in healthy individuals [1,2]. Morey and colleagues [3] built on these findings by applying a previously employed fear-conditioning paradigm, in which 50% of the exemplars of a semantic category (either animals or tools) were paired with an electric shock [1], to a sample of posttraumatic stress disorder (PTSD) patients. Such an approach that utilizes multivariate (i.e., pattern-based) analyses provides the field an additional route for developing functional neuroimaging biomarkers of anxiety disorders.

The univariate (i.e., amplitude-based) results in the article partially echo the authors’ expectations (in that PTSD patients yielded a higher signal change for the conditioned category, compared to the neutral category, with respect to trauma-exposed veteran controls) in brain regions previously linked to fear learning [4]. However, the authors additionally expected similar univariate effects and increased pattern similarity in category-selective regions (in the occipitotemporal cortex) for the PTSD patients (ostensibly linked to hyper-reactivity of the salience network), which, interestingly, they did not find. Instead, the dissimilarity matrices displayed in the authors’ fourth supplementary figure suggest that any within-category similarity increases in the PTSD group may be negligible.

The effect sizes of these multivariate results appear dramatically reduced (for both the PTSD patients and controls) compared to previously reported within-category similarity increases for healthy individuals in the occipitotemporal cortex [1]. Coupling this deviation with the authors’ prediction of *stronger* pattern-based effects in the patients raises the question: is it not the unexpected nature of the results that renders them even more interesting? Such results suggest a potential distinction in how fear-generalization mechanisms may interact with information processing in fronto-limbic as opposed to occipitotemporal regions when measured with amplitude- versus pattern-based methods; thus, placing null findings into a larger context can foster new theories.

Moreover, that the authors’ multivariate results do *not* show strong generalization within category-selective cortex raises the possibility that the cohort investigated may meaningfully differ from individuals with, for example, no combat exposure or even lower scores on trauma/depression-related questionnaires. As the authors discuss, one might find the “expected” effects in category-selective brain regions when using more ecologically valid (or pertinent) stimuli for PTSD patients. However, turning the argument around leads to the idea that, despite the behavioral results, generalization mechanisms linked to category-selective cortex may *fail to engage* in trauma-exposed individuals (at least with non-pertinent stimuli); the specifics of such a phenomenon could spark a promising new line of research.

While the authors understandably focus their discussion on the positive results, one should not gloss over the unexpected multivariate results, as they may indicate cognitive differences underlying individuals with PTSD, subclinical PTSD, or a general susceptibility to anxiety disorders (potentially contributing to further subtyping [5]). Ultimately, revolutionizing psychiatry research with functional neuroimaging will require breaking away from traditional expectations and cleverly translating cognitive neuroscience methods (like those employed by Morey and colleagues) to the clinical domain.
